# Reduced Neural Distinctiveness of Speech Representations in the Middle-Aged Brain

**DOI:** 10.1162/nol_a_00169

**Published:** 2025-06-18

**Authors:** Zhe-chen Guo, Jacie R. McHaney, Aravindakshan Parthasarathy, Kailyn A. McFarlane, Bharath Chandrasekaran

**Affiliations:** Roxelyn and Richard Pepper Department of Communication Sciences and Disorders, Northwestern University, Evanston, IL, USA; Department of Communication Science and Disorders, University of Pittsburgh, Pittsburgh, PA, USA

**Keywords:** cortical speech processing, electroencephalography, middle-aged adults, neural dedifferentiation, phoneme representation

## Abstract

Speech perception can decline in middle age even when hearing thresholds remain normal, and the underlying neurobiological mechanisms are not well understood. In line with the age-related neural dedifferentiation hypothesis, we predicted that middle-aged adults show less distinct cortical representations of phonemes and acoustic-phonetic features relative to younger adults. In addition to an extensive audiological, auditory electrophysiological, and speech perceptual test battery, we measured electroencephalographic responses time-locked to phoneme instances (phoneme-related potential) in naturalistic, continuous speech and trained neural network classifiers to predict phonemes from these responses. Consistent with age-related neural dedifferentiation, phoneme predictions were less accurate, more uncertain, and involved a broader network for middle-aged adults compared with younger adults. Representational similarity analysis revealed that the featural relationship between phonemes was less robust in middle age. Electrophysiological and behavioral measures revealed signatures of putative cochlear neural degeneration (CND) and speech perceptual deficits in middle-aged adults relative to younger adults. In line with prior work in animal models, proxies of CND were associated with greater cortical dedifferentiation, explaining nearly a third of the variance in PRP prediction accuracy together with measures of acoustic neural processing. Notably, even after controlling for CND proxies and acoustic processing abilities, age-group differences in cortical PRP prediction accuracy remained. Overall, the results reveal “fuzzier” cortical phonemic representations in middle age, suggesting that age-related neural dedifferentiation may underlie speech perceptual challenges despite a normal audiogram.

## INTRODUCTION

Speech is a ubiquitous, socially relevant auditory signal that shapes spoken language communication throughout our life-span. Despite its obvious value, speech perception can decline with age independently of audibility, a change that is distinctly noticeable by middle age (Helfer & Jesse, 2021). Why do middle-aged adults show a decline in speech perception? Prior work has suggested that they may demonstrate temporal processing challenges due to age-related deafferentation of cochlear nerve synapses, referred to as peripheral cochlear neural degeneration (CND; Ruggles et al., 2012). While this condition has been histologically verified in humans and animal models (Liberman & Kujawa, 2017; Parthasarathy & Kujawa, 2018; Wu et al., 2019), its perceptual consequences are still unclear and controversial (Bramhall et al., 2019). Here, we turn the focus to more central, cortical mechanisms and test a novel premise that middle-aged adults show reduced distinctiveness and greater dedifferentiation in the cortical representations of speech sounds that contribute to speech perceptual challenges. This premise is derived from the neural dedifferentiation hypothesis of aging, which posits that age-related processing challenges are driven by reduced specialization in the sensory cortex, leading to less distinctive and more correlated activity across the cortex (Koen & Rugg, 2019; Koen et al., 2020; Park et al., 2004). Ensembles in the temporal cortex encode critical phonological features that are causally linked to the percept of speech (Engineer et al., 2008; Mesgarani et al., 2014). Notably, auditory cortical ensembles show age-related dedifferentiation and noisier processing due to induced CND in animal models (Lalwani et al., 2019; Resnik & Polley, 2021). Given these findings, in addition to assessing age-related changes in cortical representations of speech, we also asked whether cortical dedifferentiation of speech in middle-age relates to putative CND. In the next few sections, we expand on our understanding of cortical speech processing, the impact of aging on neural distinctiveness of speech, and the potential consequence of putative CND on cortical speech processing. Finally, we discuss the main hypotheses of the study.

Speech perception has been variously described as being at the interface of neurobiology and linguistics and results from an interplay between bottom-up peripheral and top-down central processes (Poeppel et al., 2008). Yet, prior work examining speech perception in middle-aged adults (Ruggles et al., 2012) have almost exclusively focused on peripheral and subcortical neurobiology and peripheral-related declines, without alluding to the possibility that some of the challenges could be driven by disruption to cortical processes that are proximal to linguistically relevant neural operations. Patterns of cortical responses to simple speech syllables (e.g., /ba/ vs. /da/) are highly correlated with behavioral discriminability of these syllables in animal models, highlighting the proximity of cortical activity to percept (Engineer et al., 2008). In real-world listening conditions, highly variable and continuous auditory signals need to be mapped to distinct, linguistically relevant, abstract phonological representations (Hickok & Poeppel, 2007). Direct intracranial recordings using electrocorticography (ECoG) have revealed that phoneme representations are an emergent property of activity in neuronal ensembles within the superior temporal gyrus (STG) which are selectively tuned to acoustic-phonetic features (Mesgarani et al., 2014). For example, acoustic onset information is preferentially encoded in the posterior STG, while regions sensitive to phonetic cues are more spatially distributed along the posterior and anterior lateral STG (Gnanateja et al., 2025; Hamilton et al., 2021; Yi et al., 2019), showing selectivity to fine-grained phonetic features such as vowel height and consonant place (Mesgarani et al., 2014). Phoneme representations emerge from the collective response patterns across these functionally distinct and anatomically interspersed local sites in the STG.

Compared with younger adults, older adults exhibit less functionally specialized and distinct cortical representations (see Koen & Rugg, 2019, for a review). This decrease in cortical specialization is manifested as reduced category-selectivity of neural responses which was originally noted in the domain of vision (Park et al., 2004). In the domain of speech perception, a functional magnetic resonance imaging (MRI) study by Du and colleagues (2016) demonstrated age-related neural dedifferentiation of simple consonant-vowel syllables, as evidenced by the finding that multivoxel pattern classifiers trained to classify consonant phonemes performed less accurately for older adults than younger adults. Older adults in the same study also showed increased recruitment of frontal motor regions, presumably to compensate for dedifferentiation of speech representations within temporal regions. Such age-related neural dedifferentiation has been primarily investigated in older adults with little focus on middle age (Helfer & Jesse, 2021). We hypothesize that dedifferentiation issues may already emerge in middle-aged adults without overt hearing loss, resulting in less differentiated cortical representations of phonemes, and may underlie speech perceptual challenges.

Crucially, CND has also been shown to negatively impact cortical auditory processing and associated perceptual discrimination. By chemically inducing CND with ouabain, studies in mice provide direct evidence that CND increased the internal noise in the auditory cortex in a way that predicted the mice’s behavioral performance on selectively detecting target sounds in background noise (Resnik & Polley, 2021). The increased cortical noise following CND suggests that deficits in the auditory periphery can be a potential contributor to dedifferentiated phoneme representations in human listeners. Therefore, in addition to examining age-related changes in cortical processing, it is also relevant to examine the extent to which measures of putative CND—along with other peripheral, speech perceptual, and behavioral measures—may collectively explain cortical phoneme differentiation.

**Figure 1.**
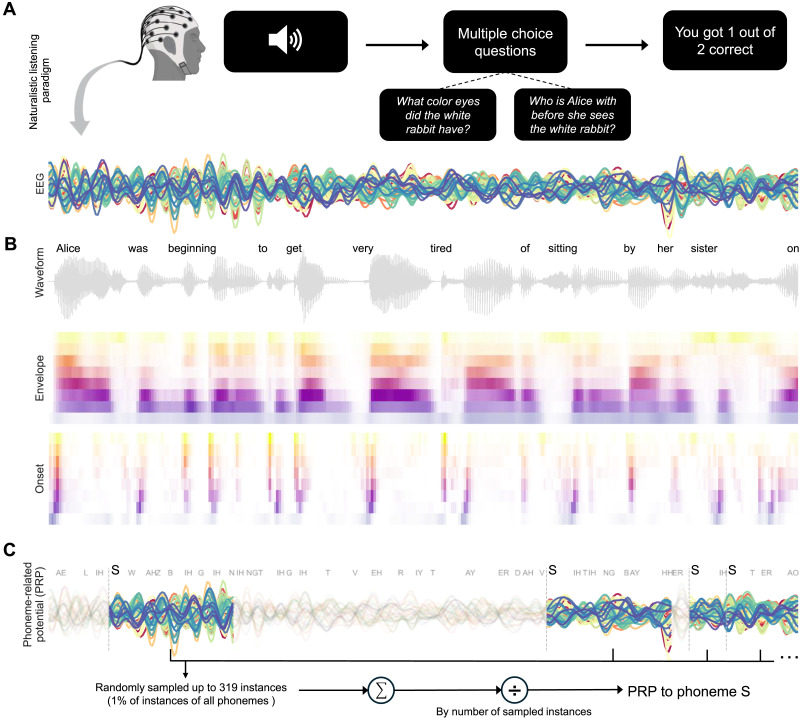
Continuous speech listening paradigm. (A) Participants listened to an audiobook version of *Alice’s Adventures in Wonderland* (Carroll, 1865/2010) in quiet while EEG activity was recorded. The figure shows preprocessed EEG responses from one participant time-aligned to an audiobook excerpt. At the end of each story segment (∼60 s), participants answered two comprehension questions regarding the content of the segment. (B) Speech waveform and 8-band envelope and onset representations of the stimuli. (C) For each participant, phoneme-related potentials (PRPs) were calculated as the averaged EEG responses from the onset of each phoneme instance (e.g., /s/) to 500 ms post-onset.

To this end, we measured electroencephalographic (EEG) responses in younger and middle-aged adults with normal hearing thresholds while they listened to continuous speech in quiet (Figure 1A). This paradigm probed the functionally and anatomically distinct response patterns in the auditory cortex evoked by continuous speech (Hamilton & Huth, 2020; Leaver & Rauschecker, 2010; Norman-Haignere et al., 2015), and a series of analyses were conducted on the EEG data. First, we computed and analyzed phoneme-related potentials (PRPs) by averaging EEG responses time-locked to phoneme instances (Figure 1C) to capture phoneme-specific neural dynamics organized by phonetic features, such as manner of articulation (Khalighinejad et al., 2017). We also measured neural tracking of key acoustic cues that are known to relate to speech perception. These include acoustic envelope and onsets (Figure 1B), the neural tracking of which could change as a function of age (Brodbeck, Presacco, et al., 2018; Gillis et al., 2023) and potentially drive differences in phoneme encoding.

Additionally, we comprehensively assessed the participants’ peripheral auditory functions, behavioral speech perception, and cognition. We examined hearing sensitivity at both standard and extended high frequencies, as elevated hearing thresholds at these frequencies can impact speech perception (Badri et al., 2011; Motlagh Zadeh et al., 2019; Yeend et al., 2019). Putative CND was assessed using proxies derived from the auditory brainstem response (ABR). Specifically, the peak-to-trough amplitudes of ABR waves I and V, which respectively reflect the summed synchronous activity of the peripheral auditory nerve and the auditory midbrain (lateral lemniscus and inferior colliculus; Hall, 2006), and wave V/I amplitude ratio. The amplitude of ABR wave I remains a consistently used proxy of CND in the literature, as it has been shown in animal models to be directly proportional to the quantity of intact synapses; that is, the amount of synaptic loss is reflected by a proportional reduction in wave I amplitude (Kujawa & Liberman, 2009; Liberman & Kujawa, 2017; Sergeyenko et al., 2013). Wave V amplitude, and further wave V/I amplitude ratio, are also suggested markers of putative CND, as unaffected or elevated wave V amplitudes or enhanced V/I amplitude ratios could indicate central gain compensation, where central auditory structures increase their response gain to compensate for the reduction in auditory input at the auditory nerve (Chambers et al., 2016; Parthasarathy & Kujawa, 2018; Schaette & McAlpine, 2011; Verhulst et al., 2016). Finally, we quantified behavioral speech perception through the Words in Noise (WIN) test, which provides less top-down context-driven scaffolding relative to other clinical speech perception tests (Wilson & Burks, 2005), and used two measures of cognitive performance to rule out any impacts of broad cognition ability on speech perception (Pichora-Fuller, 2003).

To anticipate, our findings demonstrate that cortical representations of phonemes and their featural relationship do become less distinct in middle age, highlighting the role of cortical aging in speech perception. Proxies of putative CND, namely wave I amplitudes and wave V/I ratios, were robustly associated with phoneme dedifferentiation. Yet, after controlling for these CND proxies and other predictors, such as acoustic processing abilities, middle-aged adults still exhibited less distinct, broadly distributed, and delayed phoneme representations, indicative of age-related neural dedifferentiation.

## MATERIALS AND METHODS

### Participants

Native English speakers were recruited for a larger study on speech perception in noise abilities in middle age. Here, we report data from the 44 participants who completed the continuous speech listening task from the larger speech perception study. These included 24 younger adults (22 females) aged 18–25 years (*M* = 21.417, *SD* = 2.020) and 20 middle-aged adults (12 females) aged 40–54 years (*M* = 46.050, *SD* = 4.347). All participants had (a) normal cognition, as determined by a score ≥25 on the Montreal Cognitive Assessment score (MoCA; Nasreddine et al., 2005); (b) no severe tinnitus, as self-reported via the Tinnitus Handicap Inventory (Newman et al., 1996); and (c) air conduction thresholds ≤25 dB hearing level (HL) at octave frequencies from 0.25 to 4 kHz. All participants provided written consent and received monetary compensation for their participation.

### Audiological Assessments

An otoscopic examination was first performed to ensure the ear canal and tympanic membrane were free of excess cerumen or other abnormalities. Air conduction thresholds were obtained using a MADSEN Astera audiometer, with Otometrics transducers (Natus Medical, Inc., Middleton, WI) and foam insert eartips in a sound attenuating booth. Thresholds were collected at 0.25, 0.5, 1, 2, 3, 4, and 8 kHz. We also measured extended high frequency (EHF) thresholds at 12.5 kHz using Sennheiser circumaural headphones and Sennheiser HDA 300 transducers. Most participants had binaural hearing thresholds ≤25 dB across standard test octave frequencies of from 0.25 to 8 kHz. One middle-aged participant had a single left ear threshold at 30 dB HL at 8 kHz, and one middle-aged participant had thresholds of 30- and 35-dB HL at 8 kHz. Binaural thresholds at 8 kHz for these two participants were 27.5- and 32.5-dB HL, respectively. For all other participants, binaural thresholds between 250 and 8000 Hz were ≤25 dB HL. Participants were not required to meet a particular threshold at the EHFs to participate in this experiment.

ABRs were recorded using the Intelligent Hearing Systems (Miami, FL) Duet system. To reduce intersubject variability and ensure optimal detection of ABR wave I (Bramhall, 2021; Prendergast et al., 2018), we placed the recording electrode (reference/inverting) closer to the site of neural generation by using gold-foil “tiptrodes” in the ear canal. Ground and active (non-inverting) electrodes were placed at Fpz and Fz, respectively. ABRs were collected in response to a 3 kHz tone burst while participants were seated in an electromagnetically shielded double-walled sound booth. The stimuli were presented at 86 dB nHL for 2,048 sweeps using ER-3C transducers (Etymotic Research, Elk Grove Village, IL) and sampled at 25 *μ*s at a rate of 9.3 per s with alternating polarity. ABR wave I and wave V peak-to-trough amplitudes were manually marked by a trained research assistant and confirmed by a second researcher. Any interscorer disagreements between the two raters were settled through reviewing together.

### Speech Perception in Noise Testing

We tested word-level speech perception in noise abilities using the Words in Noise task (Wilson & Burks, 2005). Testing was performed using the MADSEN Astera audiometer, with Otometrics transducers (Natus Medical, Inc., Middleton, WI) and foam insert eartips in a sound attenuating booth. The WIN stimuli were comprised of open-set single words, with no surrounding linguistic context. The WIN test list consisted of seven groupings of five words presented in four-talker babble that was at a fixed sound level of 65 dB sound pressure level (SPL). The sound level of the target word varied relative to the background noise to produce the following signal-to-noise ratio (SNR) levels: 24, 20, 16, 12, 8, 4, and 0 dB. The words were presented in descending SNR level, resulting in more difficulty as the task progressed. On each trial, the participant would hear a female speak: “Say the word _____.” Participants were instructed to repeat the target word back to the best of their ability and to make their best guess if they were unsure of what was said. Two test lists were presented, resulting in 10 target words per SNR level. The SNR loss in dB was calculated for each participant, which reflects the optimal SNR level necessary to achieve 50% accuracy, using the equation: 26 − (*n* × 0.4), where *n* reflects the sum of words correctly identified (Tillman & Olsen, 1973).

### Continuous Speech Stimuli

The stimuli were continuous speech from the public domain audiobook *Alice’s Adventures in Wonderland* (Carroll, 1865/2010), recorded at 22.05 kHz sampling rate by a male American English speaker. These stimuli have been extensively used to examine cortical speech processing across the life-span (Dial et al., 2021; Gnanateja et al., 2025; Llanos et al., 2021; McHaney et al., 2021; Reetzke et al., 2021; Xie et al., 2023). Long pauses were reduced to a maximum of 500 ms and the audiobook was partitioned into segments of ∼60 s (range: 59–65 s). Participants listened to 15 segments in each of three listening conditions (quiet, speech-shaped noise, and reversed talker babble), in counterbalanced orders with the 15 segments within each condition presented chronologically to preserve the storyline. At the end of each segment, participants answered two four-choice comprehension questions to assess their understanding of the content in the preceding segment. Continuous speech stimuli were presented binaurally through ER-3C insert earphones (Etymotic Research, Elk Grove Village, IL) at approximately 80 dB SPL in quiet conditions. The Montreal Forced Aligner (McAuliffe et al., 2017) was run to obtain word and phoneme segmentations of the stimuli. In the current study, we analyzed and reported the data from the quiet condition. Figure S1 in Supplementary Materials, available at https://doi.org/10.1162/nol_a_00169, shows the average power spectrum for each phoneme from the audiobook that was included in the PRP analysis.

### EEG Acquisition and Preprocessing

Electrophysiological responses to continuous speech were amplified and digitized with BrainVision actiCHAMP amplifier and collected using BrainVision PyCorder 1.0.7 (Brain Products, Gilching, Germany) with 64-channel actiCAP active electrodes (Brain Products) secured in an elastic cap (EasyCap; https://www.easycap.de/). Electrodes were placed on the scalp according to the International 10–20 system (Klem et al., 1999) and a common ground was placed at the AFz electrode site. Electrode impedance was less than 25 kΩ for all channels. Raw EEG responses were recorded at a 25 kHz sampling rate and preprocessed using EEGLAB (Version 14.1.2; Delorme & Makeig, 2004) in MATLAB. The data were downsampled to 128 Hz for computational efficiency, band-pass filtered from 1 to 15 Hz using minimum-phase causal windowed sinc FIR filters, and re-referenced to the average of two mastoid electrodes (Di Liberto et al., 2015, 2018; O’Sullivan et al., 2015). At the re-referenced channels, electrical activity outside the ±3 standard deviation range of the surrounding channels was rejected and interpolated, and artifacts were suppressed with artifact subspace reconstruction (ASR; Mullen et al., 2015). The ASR-cleaned data were epoched from −5 to 70 s relative to the story segment onset. Independent component analysis was performed on the epoched data to remove ocular and muscular artifacts and reconstruct the EEGs.

### EEG Data Analyses

#### Phoneme-related potential classification

We adopted the PRP approach (Khalighinejad et al., 2017) to examine cortical representations of phoneme categories (Figure 1C). For each phoneme, processed EEGs time-locked to 0.0–0.5 s after the onset of each phoneme instance were averaged to create a 61 (electrodes) × 64 (time points) PRP array representing the typical evoked response to that phoneme. As phoneme frequency distribution in natural speech is highly skewed (e.g., AH and OY accounted for about 10% and 0.08%, respectively, in our stimuli), we retained only the 31 phonemes with >319 instances (i.e., >1% of instances of all phonemes) excluding the highly frequent AH. To further minimize the possibility that phonemes with many instances could lead to high SNRs in the PRPs and bias the results, we imposed an upper limit on the number of instances included in the PRP calculation, which was set to 319. For phonemes with over 319 instances, we averaged a random subset. Subsequent analyses showed that phoneme prediction accuracy of the PRP classifier was not significantly correlated with phoneme frequency in the audiobook (*r* = −0.139, *p* = 0.456).

**Figure 2.**
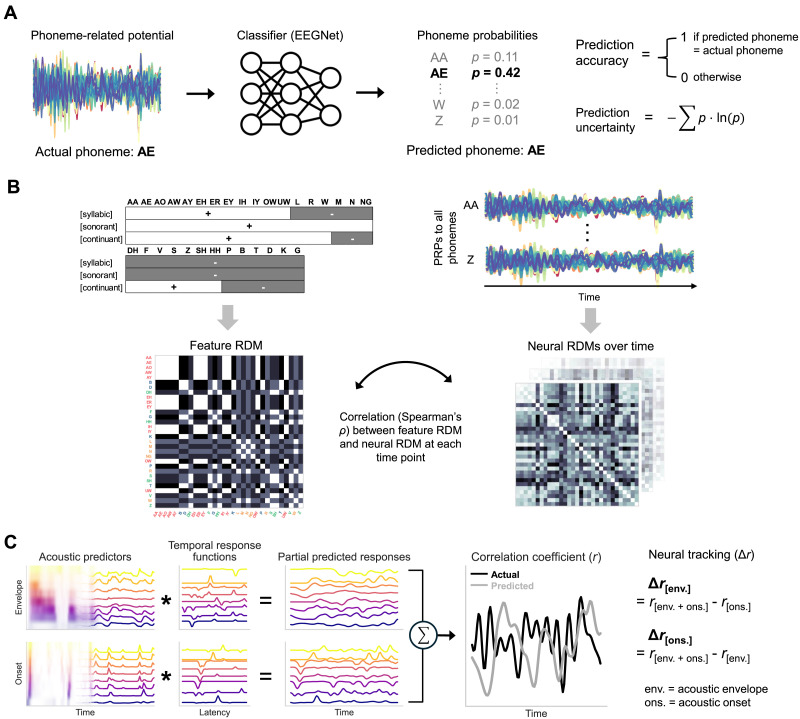
EEG data analyses. (A) EEGNet convolutional neural network classifiers were trained with cross-validation to predict the phoneme label for each held-out phoneme-related potential. The phoneme with the highest probability was recorded as the prediction and Shannon’s entropy measured the prediction uncertainty (higher entropy indicates more uncertainty). (B) Feature values of all phonemes for the features of [syllabic], [sonorant], and [continuant]. Phoneme dissimilarities based on this featural characterization were modeled as a representational dissimilarity matrix (RDM), which consisted of Euclidean distances between all pairs of phonemes. Phoneme labels in the resulting feature RDM are colored based on the manner of articulation (vowels, approximants and nasals, fricative, stops). A series of RDMs representing neural dissimilarity between phoneme pairs over time were similarly constructed from PRPs. The neural RDM at each time step was compared with the feature RDM using Spearman’s rank correlation coefficient. (C) Estimation of neural tracking of acoustic envelope and onset features. Amplitude envelope and onset envelope at each frequency band were convolved with the corresponding cross-validated temporal response functions to predict partial neural responses, which were summed to derive with the predicted response. The prediction was correlated with the actual EEG at each electrode using Pearson’s *r* as the accuracy metric. To isolate the neural tracking that could be attributed uniquely to either the envelope or onset predictor (Δ*r*), we subtracted the prediction accuracy of the model without either predictor from that of the full model including both predictors.

To assess the cortical encoding of distinct phonemes, we leveraged the EEGNet model (Lawhern et al., 2018) and trained it to predict phoneme classes from PRPs (Figure 2A). EEGNet is a compact convolutional neural network that has been demonstrated to learn useful features for classifying single-trial EEGs and event-related potentials across different brain-computer interface paradigms while allowing interpretation of the learned features. An advantage of such neural models is the ability to capture potentially nonlinear dynamics that may integrate information from multiple distributed electrodes and demonstrate phoneme representations. Here, we used the EEGNet-8,2 model with eight temporal filters, two spatial filters per temporal filter, and a dropout rate of 0.5 (Lawhern et al., 2018). We trained the model on the PRP datasets from younger and middle-aged adults separately with 20-fold cross-validation. At each iteration, 5% of the data were held out as the test set and a random 15% of the non-test data served as the validation set. The model was trained on the remaining PRPs with an initial learning rate of 0.001 and a batch size of 16 for a maximum of 300 epochs to minimize cross-entropy loss. The learning rate was decreased by a factor of 0.7 every 100 epochs. The model at the epoch with the lowest validation loss was selected to predict the test set. A prediction was correct when the highest probability phoneme matched the actual phoneme, and the prediction uncertainty was quantified as the Shannon entropy of the probabilities over all phonemes: −∑*p* · ln(*p*) (higher indicated more uncertainty).

The whole training and testing, as well as the selection of phoneme instances in PRP derivation, was repeated 20 times with different random seeds to ensure robust findings. Also, as the younger group had four more participants than the middle-aged group, we randomly dropped four younger adults at each repetition to prevent any biases due to the slightly imbalanced data. The accuracy and uncertainty results were averaged across all repetitions and phonemes for each participant and compared across the two age groups (see Statistical Tests for details). Model training was conducted using PyTorch (Version 2.1.0; Paszke et al., 2019) and the EEGNet implemented in TorchEEG (Zhang et al., 2024).

#### PRP relevance analysis

Despite the neural network’s black-box reputation, EEGNet allows interpretations of its learned representations. We leveraged this capacity of EEGNet and the Deep Learning Important FeaTures (DeepLIFT) algorithm (Shrikumar et al., 2017) implemented in Captum (Kokhlikyan et al., 2020), a model interpretability library for PyTorch, to assess the extent to which responses at a particular electrode or time point in a PRP were relevant for the trained EEGNet models to predict the target phonemes. DeepLIFT assigned a score representing the amount of evidence each input value provided toward the target phoneme by backpropagating the contributions through the network to the input. The algorithm yielded a 61 × 64 contribution score matrix for each PRP, with positive and negative values denoting evidence for and against the target phoneme, respectively, and zero indicating irrelevance. We focused on quantifying relevance regardless of the sign of evidence by taking the absolute scores and *z*-transformed the scores within each matrix to compute relative relevance. The results were averaged across phonemes and across the 20 repetitions to obtain, for each participant, a single score matrix summarizing the relevance of each electrode over time. We averaged across time and computed the variance between electrodes as a measure of relevance dispersion (smaller indicates more distributed relevance). We also averaged across electrodes to determine the timing of the relevance peak relative to PRP onset. A mass-univariate independent *t* test was run to compare the relevance topographies of the two age groups and identify clusters of electrodes showing a significant group difference.

#### Representational alignment with phonetic features

The PRP classifier treated phonemes as independent, atomic categories. Yet, as discussed, cortical phoneme representations emerge from neural ensembles selectively tuned to specific acoustic-phonetic features (Gnanateja et al., 2025; Hamilton et al., 2021; Mesgarani et al., 2014; Yi et al., 2019), providing fundamental cues to phonemes and hence speech perception. We thus further examined whether the PRPs of younger and middle-aged adults also differed in the feature-level connection between phonemes, which can be formally expressed using binary-valued distinctive features from phonological theories (Chomsky & Halle, 1968). In phonology, a phoneme can be specified with either + or − for a feature, indicating the presence or absence of that feature. The feature values collectively define the identity of a phoneme and classes of phonemes sharing similar properties. Given that cortical oscillations captured by EEG primarily reflect distinctions based on manner of articulation (Khalighinejad et al., 2017), we considered the three features that capture major manner class distinctions: [syllabic], [sonorant], and [continuant] (Figure 2B). [syllabic] indicates whether a sound can function as the syllable nucleus, separating vowels ([+syllabic]) from consonants ([−syllabic]). [sonorant] refers to sounds produced with an open vocal tract and continuous non-turbulent airflow, distinguishing obstruents ([−sonorant]) versus approximants and nasals ([+sonorant]) among consonants. [continuant] specifies whether a sound is produced with the blocking of the oral cavity and differentiates between approximants ([+continuant]) versus nasals ([−continuant]) among sonorants and between fricatives ([+continuant]) versus stops ([−continuant]) among obstruents. Note that as instances of the same phoneme generally share the same values for the three manner-defining features regardless of their position in a word or variations in acoustic realization, the features model an abstract relationship between phonemes.

We investigated the extent to which PRPs reflected such a featural relationship through the representational similarity analysis (Kriegeskorte et al., 2008; Figure 2B). The presence and absence of each feature were coded as 1 and 0, respectively, and the Python rsatoolbox library (Lin & Kriegeskorte, 2024) was used to construct a representational dissimilarity matrix (RDM) containing pairwise Euclidean distances between all phonemes. The same procedure was applied to PRPs to derive a series of neural RDMs capturing the neural dissimilarities between phonemes over time. The neural RDMs were then each compared against the phonetic-feature RDM through Spearman’s rank correlation *ρ*, with higher *ρ* suggesting better featural-neural representational alignment. As the electrodes might not all bear critical feature information, we employed a forward selection algorithm to determine the optimal set of electrodes for each age group. First, we ranked the electrodes based on the relevance scores from the PRP relevance analysis. Next, the top two most relevant electrodes were included to build neural RDMs and calculate a *ρ* value averaged over time and over participants in the group, which represented the overall fit to the feature RDM. This process iterated, each time adding the most relevant electrode from the rest of the set, until the overall *ρ* stopped improving. The optimal *ρ* curves of the younger and middle-aged listeners were compared using generalized additive modeling. RDMs were also constructed from the prediction confusion matrices of the PRP classifiers and compared with the full feature RDM using the same *ρ* metric. The observed *ρ* difference between the younger and middle-aged groups was tested using permutation analysis.

#### Estimation of neural tracking of acoustics

It was possible that our two age-groups differed in the neural tracking of continuous acoustic properties relevant to speech perception, which might explain any differences in the PRP analyses. Indeed, responses in the auditory cortex robustly track speech envelopes (Lalor & Foxe, 2010; Nourski et al., 2009) and are particularly sensitive to acoustic edges (Daube et al., 2019; Hamilton et al., 2018), and the ability to track these acoustic cues is impacted by aging (Brodbeck, Presacco, et al., 2018; Gillis et al., 2023). To address this possibility, we compared the neural tracking of acoustic envelope and onsets (Figure 1B) across the two age groups. The continuous spectro-temporal acoustic properties of the stimuli were modeled as spectrograms using gammatone filters, simulating the frequency analysis performed in the cochlea (Brodbeck et al., 2020; Patterson et al., 1992). We used the gammatone filters from the Python Eelbrain package (Brodbeck et al., 2023) with 256 filter channels and cut-off frequencies of 0.02–5 kHz. The 256-band spectrograms were downsampled to 1 kHz, scaled with an exponent of 0.6, and summed into eight logarithmically spaced frequency bands to derive 8-band gammatone envelope (spectrogram) predictors for subsequent analyses. Additionally, we controlled for representations of acoustic onsets by applying an auditory edge detection model (Fishbach et al., 2001) to the 256-band gammatone spectrograms using the parameters in Brodbeck et al. (2020). The resulting onset spectrograms were summed into eight logarithmically spaced frequency bands, creating 8-band onset spectrogram predictors.

Neural tracking of acoustics was estimated by fitting multivariate temporal response function (mTRF) models (Crosse et al., 2016) that describe the linear forward mapping from the acoustic predictors to EEGs (Figure 2C). TRFs to a continuous stimulus such as the gammatone spectrogram can be interpreted as the evoked responses to an elementary event at each frequency band of the spectrogram. We included either or both the envelope and onset spectrograms as predictors and used the boosting function in Eelbrain to derive TRFs in each electrode at time lags from −100 to 500 ms with *ℓ*_1_ error norm, following a fivefold cross-validation procedure. At each iteration, EEGs from three of the 15 story segments were held out as the test data while those from the other segments were used to train and validate TRF models, which were averaged to predict the test set when the estimation was finalized. The Pearson’s correlation coefficient (*r*) between the predicted and observed EEGs measured how accurately the brain tracked the predictor. We estimated neural tracking that could be uniquely attributed to the predictor of interest (Δ*r*) by taking the *r* difference between the full model including both spectrogram and onset spectrograms and the model with that predictor left out. Topographies of neural tracking were compared across the two age groups with a mass-univariate independent *t* test. They were also compared against zero with a mass-univariate one-sample *t* test to identify the region of interest (ROI), or electrodes with a significantly greater-than-zero Δ*r*. A single neural tracking score was obtained for each participant by average the Δ*r* values in the ROI.

### Statistical Tests

Welch’s two sample *t* tests were calculated using the rstatix package (Kassambara, 2023) in R (Version 4.3.1; R Core Team, 2023) to compare the two age groups across variables that were normally distributed. Normally distributed variables included audiological and cognitive tests, relevance dispersion, and average neutral tracking (Δ*r*) of acoustic envelope and onsets in the ROI, and accuracy of comprehension question responses. Normality was confirmed with Shapiro-Wilks tests (*p* > 0.05) and visual inspection of quantile-quantile plots. Mann-Whitney *U* tests were used to compare phoneme prediction accuracy and uncertainty values from the PRP classifier, latencies of relevance peak, and EHF thresholds at 12.5 kHz between groups because the data were not normally distributed.

For topographical data, mass-univariate *t* tests were conducted using Eelbrain to compare variables of interest against zero (one-sample tests, one-tailed) or between younger and middle-aged adults (independent-samples tests, two-tailed). The mass-univariate *t* test is a cluster-based permutation test that uses a *t* value equivalent to uncorrected *p* ≤ 0.05 as the cluster forming threshold. Clusters were based on the identification of meaningful effects across groups of adjacent electrodes that showed the same effect (Maris & Oostenveld, 2007). A corrected *p*-value was then computed for each cluster based on the cluster-mass statistician null distribution from 10,000 permutations (Maris & Oostenveld, 2007). The largest absolute *t*-value from the cluster (*t*_max_) is reported as an estimate of effect size (Brodbeck, Hong, & Simon, 2018). The tests were used to compare the topographical scalp maps of neural tracking of the acoustic predictors and the time-varying relevance scores (analysis time window: 0 to 500 ms). A mass-univariate *t *test was also run on the data from all participants to identify the ROI for acoustics tracking, which was the set of electrodes with tracking scores significantly greater than zero.

Generalized additive models using the mgcv package (Wood, 2021) in R were performed to identify age-group differences in time series data, including the *ρ* values from the RDM analyses and the average beta weights in the ROI from the acoustic tracking mTRFs. The models consisted of the constant difference between the two groups and the smooth term s(time, by = group) that captured potential nonlinearity over time while allowing the degree of nonlinearity to vary across groups. A smooth term, s(time, subject, bs = “fs”, m = 1), was also included to allow differing degrees of nonlinearity across participants. We plotted the estimated group difference curve and identified the interval during which the difference was significant (when 95% confidence interval excluded zero).

The permutation analysis for the observed group difference in *ρ* from the RDM analyses was conducted by randomly shuffling the rows of the confusion matrices, computing a new *ρ* difference, and repeating the process for 5,000 times to build a null distribution of differences. The proportion of difference values smaller than the observed one was treated as the *p* value.

Finally, we performed a backward stepwise regression to determine which factors explained significant variance in phoneme prediction accuracy of the PRP classifier. Because regressions have no non-parametric alternatives, variables violating the normality assumption were transformed. Phoneme prediction accuracy was converted to rationalized arcsine units (RAU; Studebaker, 1985) to normalize the distribution and mitigate floor and ceiling effects. EHF thresholds at 12.5 kHz were logarithmically transformed to also meet normality after adding 25 to all thresholds to ensure no negative or non-zero values. Then, two multiple regressions were fit using the lm function in lmerTest (Kuznetsova et al., 2017) with RAU phoneme prediction accuracy as the outcome variable. The first regression contained predictor variables of ABR wave I amplitudes, ABR wave V amplitudes, pure tone average (PTA) across 1, 2, and 4 kHz, transformed EHF thresholds at 12.5 kHz, neural tracking of acoustic onsets, neural tracking of acoustic envelope, operation span task (OSPAN) scores, and MoCA scores. To avoid issues from high multicollinearity between ABR wave I and V amplitudes with wave V/I amplitude ratio, a second regression was performed, which contained predictor variables of ABR wave V/I ratios, PTA across 1, 2, and 4 kHz, transformed EHF thresholds at 12.5 kHz, neural tracking of acoustic onsets and acoustic envelope, OSPAN, and MoCA. All predictor variables were *z*-scored to allow for better interpretation of the standardized regression coefficients. The backward stepwise regressions were performed using the stepAIC function in the MASS package (Venables & Ripley, 2002) in R. It removed a predictor variable at each step to find the combination of variables that resulted in a model with the lowest Akaike Information Criteria (AIC). The predictors in the best fit models according to the stepwise regressions were then included in separate multiple regression models using the lm function to predict RAU phoneme prediction accuracy. The residuals function was used to extract the residuals in accuracy for each model; these results were then subjected to a Welch’s *t* test, based on normality of variables, to determine whether the two age groups differed in phoneme prediction accuracy after factoring out the impact of those predictors.

## RESULTS

### Middle-Aged Adults Showed Less Neural Distinctiveness of Phoneme Encoding Than Younger Adults

**Figure 3.**
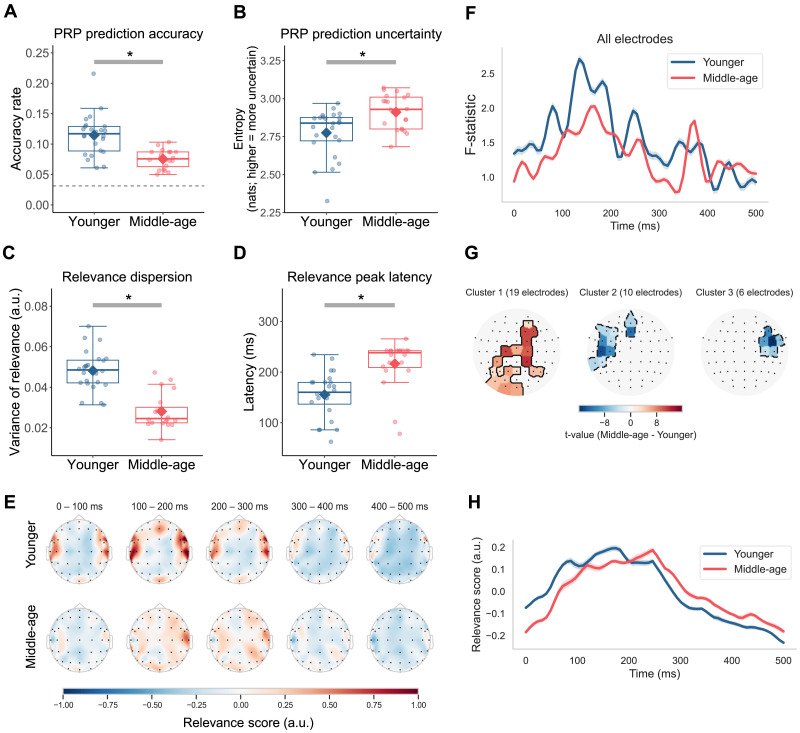
Less distinct phoneme encoding in middle-aged adults. (A–D) Boxplots of phoneme prediction accuracy, prediction uncertainty, relevance dispersion, and relevance peak latency for the younger and middle-aged adults, with individual participants’ values (dots) and the group mean (diamond). Boxplots show the median (horizontal line), 25th and 75th percentiles (box edges), and ±1.5 interquartile range (whiskers). Asterisks indicate statistical significance at the 0.05 level. Dashed horizontal line in the accuracy panel marks the chance-level performance. (E) Topographies of averaged relevance score in 100-ms time bins over the duration of PRP. (F) *F* statistic of the PRPs over time calculated using all electrodes. Ribbons around the curve represent ±1 standard error. (G) Clusters of electrodes with significant group difference (*p* < 0.05, two-tailed) based on mass-univariate independent-samples *t* tests comparing relevance scores (averaged over time) between younger and middle-aged adults. Electrodes are color-coded for *t* values. (H) Time course of relevance averaged over all electrodes and participants in each age group. Ribbons around the curve represent ±1 standard error.

Response accuracy of the comprehension questions during the listening task did not differ significantly between younger (*M* = 0.872, *SD* = 0.083) and middle-aged (*M* = 0.867, *SD* = 0.090) adults (Welch’s *t*(39.102) = −0.276, *p* < 0.784, 95% CI [−0.060, 0.046]). However, neural responses to phonemes were less distinct for the middle-aged group, as confirmed by the results of the classifier trained to predict phoneme labels from PRPs. While the median phoneme prediction accuracy for both age groups was significantly above the chance level of 3.23% (younger: *z* = 4.286, *p* < 0.001; middle-aged: *z* = 3.925, *p* < 0.001), phoneme prediction accuracy of middle-aged adults (*Mdn* = 0.075) was significantly lower than that of younger adults (*Mdn* = 0.117; *z* = −4.151, *p* < 0.001; Figure 3A). Consistently, the prediction uncertainty of the classifier, measured as Shannon’s entropy, was significantly greater for middle-aged adults (*Mdn* = 2.930) than for younger adults (*Mdn* = 2.839, *z* = 2.569, *p* = 0.010; Figure 3B). These results agreed with an additional analysis that quantified neural separability of phonemes by computing the *F* statistic on the PRPs over time (Khalighinejad et al., 2017; Figure 3F). Here, the *F* statistic was the ratio of between-phoneme variability over within-phoneme variability, with higher values indicating greater category separation. Throughout the duration of the PRP, the *F* statistic was generally higher for younger adults (*M* = 1.540, average *SD* = 0.472) than for middle-aged adults (*M* = 1.314, average *SD* = 0.316), especially during the first 350 ms. Together, these findings converge to suggest that neural representations of phonemes are less distinct or separable in middle age.

### Middle-Aged Adults’ Phoneme Processing Involved a More Distributed Network With a Delayed Timing

We next investigated the extent to which spatiotemporal features of the PRPs were relevant to phoneme predictions and whether the patterns differed between the two age groups. This analysis was enabled by the DeepLIFT algorithm (Shrikumar et al., 2017), whereby we derived relevance scores summarizing the degree to which each electrode contained information relevant to predicting the target phoneme, with higher values representing greater relevance.

Topographic maps of average relevance scores in 100-ms intervals over the duration of the PRP for each age group are depicted in Figure 3E. One immediate observation was that the relevance was more localized for younger adults but more uniformly distributed for middle-aged adults. To test this observation, we averaged relevance scores over time for each electrode and computed relevance dispersion, or the variance of these scores between the electrodes, for each participant. Relative to that of the younger group (*M* = 0.048, *SD* = 0.010), the relevance dispersion of the middle-aged group was significantly smaller (*M* = 0.028, *SD* = 0.009; Welch’s *t*(41.851) = −7.213, *p* < 0.001, 95% CI [−0.026, −0.014]), suggesting that relevance values across electrodes were more similar, and hence, that important information for phoneme identity was more spatially distributed (Figure 3C). A mass-univariate independent *t* test was conducted to specifically identify which electrodes showed significant group differences. The results revealed three clusters of electrodes (Figure 3G), including one large cluster spanning electrodes from the occipital to fronto-central area with higher relevance scores for middle-aged adults (*t*_max_ = 11.573, *p* < 0.001). The other two smaller clusters included mostly temporal electrodes in the left (*t*_max_ = −13.947, *p* < 0.001) and right (*t*_max_ = −15.854, *p* < 0.001) hemisphere with lower relevance for the middle-aged group.

Furthermore, we compared the temporal dynamics of relevance scores across both age groups. For each participant, we averaged relevance over all electrodes at each time point in the PRP to derive a time-varying relevance curve (Figure 3H) and identified the latency at which the relevance value reached its maximum. The relevance peak latencies of middle-aged adults (*Mdn* = 238.281) were delayed relative to those of younger adults (*Mdn* = 160.156; *z* = 4.208, *p* < 0.001; Figure 3D). These results indicated that phoneme processing in middle age may be less efficient and supported by a relatively broader cortical network.

### Reduced Encoding of Featural Relationship Between Phonemes in Middle Age

**Figure 4.**
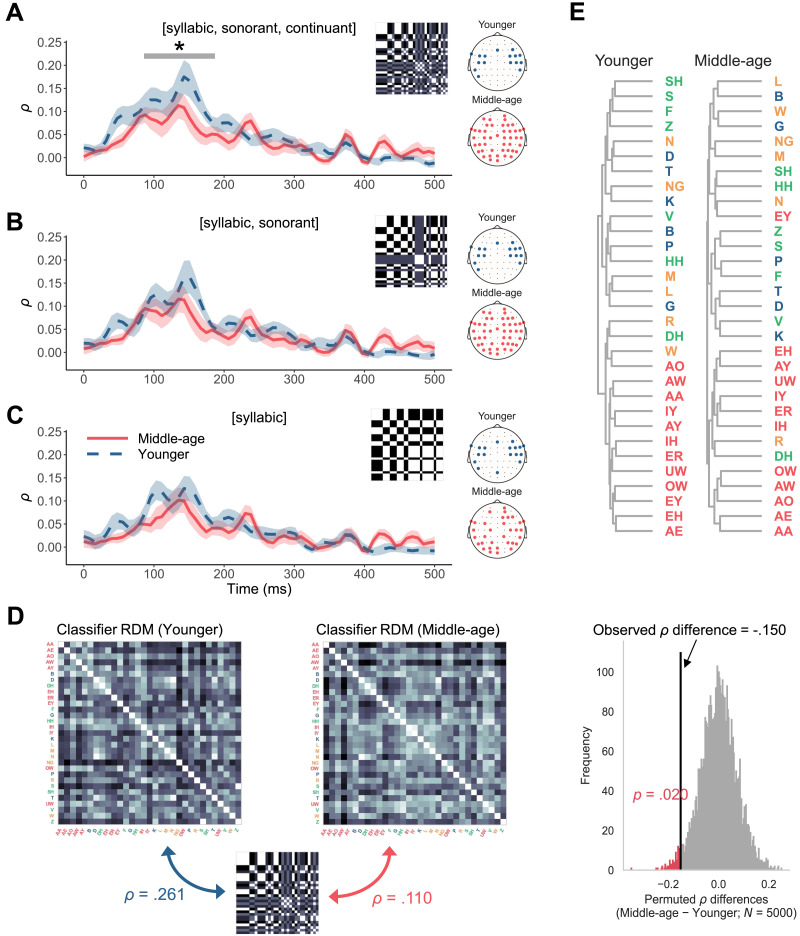
Less robust featural relationship between phonemes in middle-age. (A–C) Average Spearman’s rank correlation *ρ* of each age group over time with ribbons indicating ±1 standard error. The correlation measured the alignment between neural representational dissimilarity matrices (RDMs) derived from phoneme-related potentials (PRPs) and a phonetic-feature RDM. Each panel presents the results for a different level of feature distinction. The topographies on the right show the electrodes included to build the neural RDMs, which were those leading to optimal alignment (highest overall *ρ*) with the RDM of each feature hypothesis. (D) RDMs were derived from the confusion matrices of the PRP classifiers and compared with the feature RDM including [syllabic], [sonorant], [continuant] for each age group. The *ρ* difference between the younger and middle-aged groups was significant according to a permutation analysis which calculated a permuted *p* value from a null distribution of *ρ* differences. (E) Hierarchical clustering of the phonemes for each age group using PRPs during the 0–350 ms time window and electrodes in panel A.

The less differentiated speech representations in middle age might also be manifested at the level of phonetic features. To test this, we investigated the extent to which the PRPs aligned with a featural description of the phonemes by deriving neural RDMs from PRPs and comparing them against a feature RDM. For both age groups, the neural RDMs showed an alignment (correlation coefficient *ρ*) peak at approximately 150 ms after phoneme onset (Figure 4A), similar to the timing of the phoneme separability *F*-statistic peak (Figure 3F). Crucially, the correlation curve was significantly lower for middle-aged adults between 86 and 187 ms, suggesting poorer agreement with the feature RDM. We additionally tested two reduced feature RDMs with [syllabic] and [sonorant] and with only [syllabic] (Figure 4B–C). This time there was no significant age-group difference when using either reduced model. The findings suggested that cortical representations of phonemes are less distinct at the featural level in middle-aged listeners. It is also worth noting that the number of electrodes included to build the neural RDMs (topomaps on the right in Figure 4A–C), which formed the set leading to the highest *ρ*, was greater for middle-aged adults than for younger adults. Such a pattern indicated that information from a broader network of electrodes was required to achieve optimal alignment with the feature-based phoneme representations, consistent with the more distributed relevance from the previous analysis (Figure 3E).

The group difference in alignment with the featural representations of the phonemes was similarly reflected in the confusion patterns of the PRP classifiers. The confusion matrices of the PRP classification from each group were used to construct RDMs, which were again compared with the full feature RDM containing [syllabic], [sonorant], and [continuant] (Figure 4D). The correlation was higher for younger adults (*ρ* = 0.261) than for middle-aged adults (*ρ* = 0.110), and this difference was significant according to a null distribution of *ρ* differences derived through permutation. Thus, not only was the encoding of individual phonemes less distinct in middle-age, but also their featural relationship was less preserved.

The results thus far are in line with the hypothesized cortical dedifferentiation of phonemes in middle age. Meanwhile, we assume that the dedifferentiation may at least partially relate to (1) reduced neural tracking of critical acoustic features associated with speech intelligibility, and (2) putative markers of CND. In the next sections, we examined these possibilities. Finally, to obtain a more holistic view of the age-related changes and the interplay between peripheral and central processes, we examined the extent to which these variables explain the neural differentiation of phoneme information.

### No Evidence for Age-Group Difference in the Neural Tracking of Acoustic Envelope and Onsets

**Figure 5.**
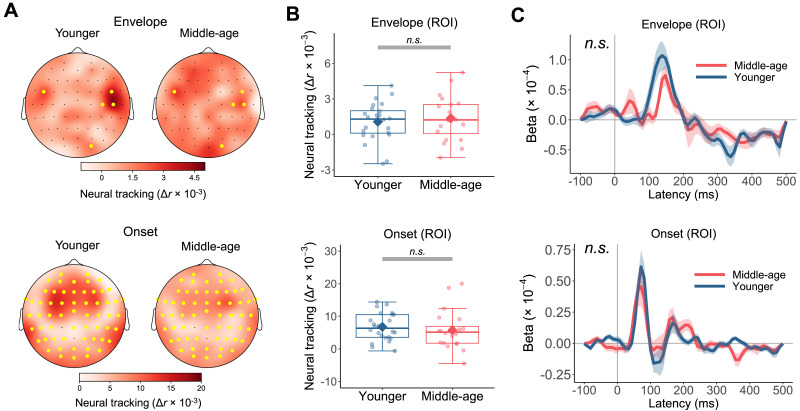
Multivariate temporal response function (mTRF) analysis provided no evidence that neural tracking of acoustic envelope and onsets differed between younger and middle-aged adults. (A) Topography of average neural tracking of envelope and onsets by age group. The region of interest (ROI; yellow dots) was defined as the set of electrodes with neural tracking significantly above zero (*p* < 0.05, one-tailed) based on mass-univariate one-sample *t* tests including all participants and collapsing across age groups. (B) Individual participants’ neural tracking scores averaged across electrodes in the ROI (dots) and the mean of each age group (diamond). Boxplots show the median (horizontal line), 25th and 75th percentiles (box edges), and ±1.5 interquartile range (whiskers). No significant age-group difference in neural tracking of acoustic envelope and onsets was found. (C) Beta weights of TRFs averaged across frequency bands, ROI electrodes, and participants in each group for each acoustic predictor. Ribbons represent ±1 standard error. TRF time courses did not differ significantly between the two groups.

A more parsimonious, acoustic-based explanation for the age-group differences observed above was that middle-aged listeners were simply less capable of tracking critical continuous acoustic properties, such as acoustic envelope and onsets. This view, however, was not supported by the mTRF analysis assessing neural tracking of acoustics. Figure 5A depicts the average neural tracking across the scalp for each age group. Mass-univariate independent-samples *t* tests suggested that there were no clusters of electrodes with significant age-group difference in the neural tracking for both the envelope (*t*_max_ = 2.372, *p* = 0.616) and onset (*t*_max_ = 2.523, *p* = 0.380) predictors. Even when considering only the electrodes in the ROI (yellow dots in Figure 5A), defined as those with neutral tracking significantly above zero, we still found no evidence that the neural tracking differed between the younger group (envelope: *M* = 1.067 × 10^−3^, *SD* = 1.612 × 10^−3^; onset: *M* = 6.741 × 10^−3^, *SD* = 4.219 × 10^−3^) and middle-aged group (envelope: *M* = 1.367 × 10^−3^, *SD* = 1.874 × 10^−3^; onset: *M* = 5.730 × 10^−3^, *SD* = 5.990 × 10^−3^), for both acoustic envelope (Welch’s *t*(37.791) = 0.562, *p* = 0.578, 95% CI [−7.794 × 10^−4^, 1.378 × 10^−3^]) and onsets (Welch’s *t*(33.257) = −0.635, *p* = 0.530, 95% CI [−4.250 × 10^−3^, 2.228 × 10^−3^]; Figure 5B).

To further test if the two groups differed in the temporal dynamics of neural responses to the acoustic features, we extracted the average beta weights of the TRFs in the ROI over time from the full model with both the onset and envelope predictors (Figure 5C). The average TRF corresponding to the acoustic onsets demonstrated an abrupt peak between 0 and 100 ms followed by a shallow valley, while that of the envelope showed a less defined and more delayed peak. Similar TRF characteristics were observed in previous studies using these acoustic representations (Brodbeck, Presacco, et al., 2018; Gillis et al., 2023). Crucially, we found no significant difference between younger and middle-aged listeners in the TRF curves either. Together, these results provided no evidence that envelope-based acoustic features were represented better or worse in middle-aged adults’ cortical responses.

### Middle-Aged and Younger Adults Show Differences in Speech Perception and Peripheral Auditory Health But Not Cognitive Performance

**Figure 6.**
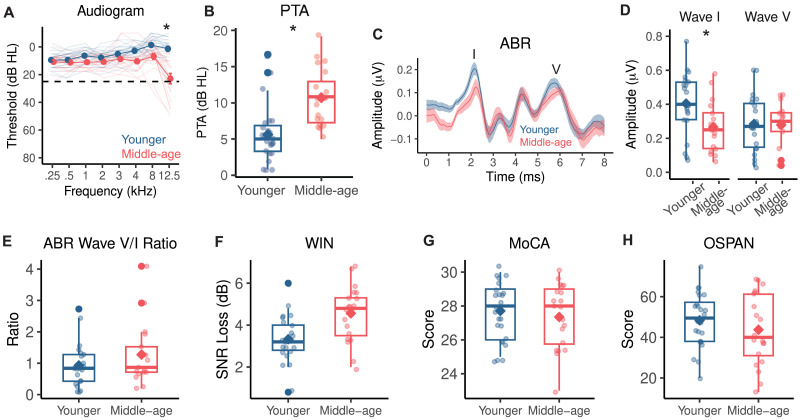
Results from audiological assessments, speech perception in noise performance, and cognitive tests. (A) Hearing thresholds (dB HL) at frequencies from 0.25 to 12.5 kHz of individual participants (transparent lines) and group averages (opaque lines). Error bars reflect standard error of the mean. Extended high frequency thresholds at 12.5 kHz were significantly higher in middle-aged adults. (B) Pure tone averages (PTA) across 1, 2, and 4 kHz were significantly higher in middle-aged adults. (C) Average auditory brainstem response (ABR) waveforms with the shaded region reflecting standard error of the mean. Wave I and wave V are marked by the I and V, respectively. (D–H) Boxplots of individual ABR wave I and wave V amplitudes, ABR wave V/I ratios, Words in Noise (WIN) signal-to-noise ratio loss (SNR) in decibels (dB), Montreal Cognitive Assessment (MoCA) scores, and operation span task (OSPAN) scores for younger and middle-aged adults. Individual participants’ values (dots) and the group mean (diamond) are depicted. Boxplots show the median (horizontal line), 25th and 75th percentiles (box edges), and ±1.5 interquartile range (whiskers). Asterisks indicate statistical significance at *α* = 0.05 level.

Speech processing requires a complex interaction between precise auditory encoding and cognitive resource utilization (McHaney et al., 2024; Parthasarathy et al., 2020; Pichora-Fuller et al., 2016; Winn et al., 2015; Zink et al., 2024). To understand whether our age groups differed on either of these domains, we looked beyond the EEG data and inspected measures from a series of audiological and cognitive tests (Figure 6). Even though all participants were considered to have clinically normal thresholds (Figure 6A), middle-aged adults (*M* = 10.708, *SD* = 3.976) had significantly higher PTAs across 1, 2, and 4 kHz than younger adults (*M* = 5.625, *SD* = 3.918; Welch’s *t*(40.361) = −4.251, *p* < 0.001, *d* = −1.288, 95% CI [−7.500, −2.667]; Figure 6B). Notably, behavioral thresholds at 3 kHz, the frequency of the tone burst stimulus used to evoke ABRs in this study, were not different between younger adults (*M* = 5.125, *SD* = 5.548) and middle-aged adults (*M* = 8.750, *SD* = 7.968; *t*(33.005) = −1.717, *p* = 0.076, *d* = −0.528, 95% CI [−7.920, 0.670]). We also measured EHF thresholds at 12.5 kHz, which is not commonly collected on the standard audiogram but is a marker for lifetime acoustic exposure that is associated with higher risk of putative CND (Liberman et al., 2016). Middle-aged adults exhibited elevated EHF thresholds at 12.5 kHz (*Mdn* = 15.00), which was statistically greater than the thresholds for younger adults (*Mdn* = −2.500, *z* = −4.559, *p* < 0.001). These findings suggested that our middle-aged adults showed some degree of subclinical hearing loss at thresholds less than 4 kHz and that they may be at a higher risk for putative CND.

Using the ABR, we probed putative CND in our participants by examining wave I and wave V peak-to-trough amplitudes and wave V/I amplitude ratio. Middle-aged adults (*M* = 0.266, *SD* = 0.152) showed significantly lower wave I amplitudes compared to younger adults (*M* = 0.404, *SD* = 0.184; Welch’s *t*(35.979) = 2.536, *p* = 0.016, *d* = 0.819, 95% CI [0.028, 0.249]; Figure 6C–D), indicating poorer peripheral auditory nerve integrity and providing evidence supporting the presence of CND in this age group. In contrast, wave V amplitudes were not different between middle-aged (*M* = 0.279, *SD* = 0.130) and younger (*M* = 0.284, *SD* = 0.164) adults (Welch’s *t*(36.963) = 0.112, *p* = 0.912, *d* = 0.036, 95% CI [−0.090, 0.101]), illustrating structures in the auditory midbrain are responding similarly across groups despite the differences in sensory input from the peripheral auditory nerve. However, the ratios of wave V/I amplitudes were not different between middle-aged (*Mdn* = 0.868) and younger (*Mdn* = 0.839) adults (*z* = −0.866, *p* = 0.387; Figure 6E). Taken together, these findings suggest that while some central gain compensation could be occurring in middle-aged adults, the degree of compensation was not different relative to younger adults.

One of the primary perceptual complaints of putative CND is difficulty understanding speech in noisy environments. To measure speech perception in noise performance, we administered the WIN test, which is commonly used in audiology clinics to measure word-level perception in multi-talker babble. The SNR loss, a metric of the SNR level required for accurate perception of words in background noise 50% of the time, was significantly higher in middle-aged adults (*M* = 4.505, *SD* = 1.339) compared to younger (*M* = 3.426, *SD* = 1.187) adults (Welch’s *t*(37.302) = −3.245, *p* = 0.002, *d* = −1.007, 95% CI [−2.002, −0.463]; Figure 6F). This indicated that the middle-aged adults in our study required quieter listening environments for accurate perception of words in noise than younger adults.

While we observed several age group differences in audiological measurements, we did not observe differences in cognitive performance. All participants completed the MoCA (Nasreddine et al., 2005) as part of a prescreening measure and were required to score higher than 24 to participate in the study. Middle-aged (*M* = 27.350, *SD* = 1.954) and younger (*M* = 27.708, *SD* = 1.706) adults had comparable MoCA scores (Welch’s *t*(38.106) = 0.641, *p* = 0.525, *d* = 0.195, 95% CI [−0.773 1.489]; Figure 6G), indicating normal cognitive function in both groups. Working memory, a cognitive process that is heavily involved in speech perception in noise, allows the listener to hold onto information, fill in missing phonemes and words, and continue to comprehend the speech stream (Rudner et al., 2011). We administered the OSPAN task (Unsworth et al., 2005) as a metric of working memory and executive functioning in our participants. As with the MoCA, we did not observe a significant difference in OSPAN scores between middle-aged (*M* = 43.750, *SD* = 17.586) and younger (*M* = 48.375, *SD* = 13.318) adults (Welch’s *t*(34.914) = 0.967, *p* = 0.340, *d* = 0.297, 95% CI [−5.081, 14.331]; Figure 6H). Taken together, these tests suggested that our age groups were cognitively matched and that the observed differences in WIN and phoneme prediction accuracy from the PRP results may be driven at least partly by putative CND rather than cognitive processing.

### Putative CND and Neural Tracking of Acoustics Explain Significant Variance in Phoneme Prediction Accuracy

Given the multitude of factors that can impact speech perception abilities, we probed the potential combined contributions of auditory and cognitive factors to cortical differentiation of phonemes. Specifically, we used a backward stepwise regression to calculate the variance in individual participants’ phoneme prediction accuracy from the PRP classification that could be explained by ABR wave I and wave V amplitudes, PTA, EHF thresholds at 12.5 kHz, neural tracking of acoustic envelope and onsets, OSPAN scores, and MoCA scores. The full regression model with all of the above listed predictors was not statistically significant (*F*(8, 28) = 2.289, *p* = 0.050), with an AIC of 109.73, and explained 22.3% of the variance in phoneme prediction accuracy. The results from the backward stepwise regression revealed that the combination of predictor variables that provided the lowest model AIC (AIC = 103.29) were ABR wave I amplitudes and neural tracking of acoustic envelope and acoustic onsets. This best fit model was statistically significant (*F*(3, 34) = 5.679, *p* = 0.003) with an adjusted *R*^2^ of 0.275, explaining approximately 28% of the variance in phoneme prediction accuracy.

Interestingly, neural tracking of acoustic onsets (*β* = 1.830, *SE* = 0.808, *t* = 2.265, *p* = 0.030) and ABR wave I amplitudes (*β* = 2.461, *SE* = 0.655, *t* = 3.755, *p* = 0.001) were statistically significant in the best fit model, suggesting that these two variables strongly contributed to phoneme prediction accuracy. Moreover, ABR wave I amplitudes had the largest coefficient, which not only indicated that ABR wave I amplitudes were the most important variable in explaining variance in phoneme prediction accuracy, but that smaller ABR wave I amplitudes were associated with lower phoneme prediction accuracy. This finding also provides evidence to suggest that participants with putative CND had noisier neural representations of phonemes, in line with the hypothesized contribution of putative CND to phoneme dedifferentiation. Neural tracking of the acoustic envelope (*β* = 1.583, *SE* = 0.784, *t* = 2.020, *p* = 0.051) was not a statistically significant predictor in the best fit model, but removing neural tracking of acoustic envelope resulted in a larger AIC (AIC = 106.13), indicating a poorer model fit without it.

We also investigated whether the relative normality of the ABR wave V amplitudes through the ABR wave V/I ratio may explain phoneme prediction accuracy to a greater extent than the individual amplitudes of wave I and wave V independently. To this end, we built a second backward stepwise regression that was identical to the previous one with the addition of the ABR wave V/I ratio, except we removed wave I and wave V as the ratio showed high multicollinearity with wave I (*r* = −0.59) and wave V (*r* = 0.62) amplitudes. The full regression model with all predictors was not statistically significant (*F*(7, 24) = 2.175, *p* = 0.074), with an AIC of 98.46, and explained 21% of the variance in phoneme prediction accuracy. The results from the backward stepwise regression indicated that the combination of ABR wave V/I ratio, PTA, amplitudes and neural tracking of acoustic envelope and acoustic onsets provided the lowest model AIC (AIC = 93.83). The intercept (*β* = 5.909, *SE* = 0.653, *t* = 9.055, *p* < 0.001) and PTA (*β* = −1.357, *SE* = 0.649, *t* = −2.090, *p* = 0.044) were the only predictors to show statistical significance. However, the combination of ABR wave V/I ratio (*β* = −1.252, *SE* = 0.699, *t* = −1.791, *p* = 0.082), PTA, and neural tracking of acoustic onsets (*β* = 1.515, *SE* = 0.841, *t* = 1.802, *p* = 0.081) and envelope (*β* = 1.163, *SE* = 0.800, *t* = 1.454, *p* = 0.155) yielded a statistically significant model (*F*(4, 33) = 3.31, *p* = 0.022) with an adjusted *R*^2^ of 0.200, explaining 20% of the variance in phoneme prediction accuracy.

In comparing the two stepwise regressions, the first regression with ABR wave I amplitudes (∼28%) explained greater variance in phoneme prediction accuracy than the second regression with ABR wave V/I ratios (∼20%). Taken together, these findings indicated that a combination of peripheral and central factors contributed to the neural representations of phonemes during continuous speech listening.

### Group Differences in Phoneme Prediction Accuracy Remained After Accounting for ABR Wave I Amplitudes and Neural Tracking of Acoustic Features

Given the significant explanatory power of the predictors from the best fit regression models, we tested whether the age-group difference in phoneme prediction accuracy (in RAU) remained after controlling for these predictors. We addressed this with a residual analysis, in which we first ran a multiple linear regression model to predict phoneme accuracy using the independent variables in the first best fit model: ABR wave I amplitudes, neural tracking of acoustic envelope, and neural tracking of acoustic onsets. The residuals (i.e., differences from actual phoneme accuracy not explained by the predictors) were extracted and compared across age groups. The results showed that phoneme prediction accuracy was still significantly lower for middle-aged adults (*M* = −2.172, *SD* = 1.909) compared with younger adults (*M* = 1.758, *SD* = 3.816; Welch’s *t*(30.614) = 4.124, *p* < 0.001, *d* = 1.302, 95% CI [1.985, 5.874]). We then ran an additional residual analysis to control for the independent variables identified in the second best fit model: ABR wave V/I ratios, PTA, neural tracking of acoustic envelope, and neural tracking of acoustic onsets. When controlling for these variables, middle-aged adults (*M* = −2.009, *SD* = 2.156) still showed significantly lower phoneme prediction accuracies than younger adults (*M* = 1.626, *SD* = 4.071; Welch’s *t*(31.529) = 3.526, *p* = 0.001, *d* = 1.116, 95% CI [1.534, 5.736]). Therefore, although putative CND, as suggested by smaller ABR wave I amplitudes, significantly predicted phoneme encoding fidelity, this and the other predictors, including PTA and ABR wave V/I ratios, could not fully explain the age-group difference in phoneme prediction accuracy.

## DISCUSSION

We recorded EEG responses to naturalistic speech from younger and middle-aged listeners with normal audiometric thresholds and assessed their cortical encoding of speech sound categories (Figure 1). By training classifiers to predict phonemes from PRPs (i.e., average EEGs time-locked to phoneme instances), we found that phonemes were predicted significantly less accurately and with greater uncertainty for middle-aged adults than for younger adults (Figure 3). Information in the PRPs relevant to phoneme predictions was more broadly distributed across electrodes and showed a delayed peak timing in the middle-aged group (Figure 3). Also, middle-aged adults’ PRPs aligned less with a phoneme representation based on the [syllabic], [sonorant], and [continuant] features, suggesting a less specified featural relationship between phonemes (Figure 4). Middle-aged adults also showed lower speech perception in noise performance (WIN score) and more pronounced peripheral signatures of putative CND (Figure 6). The proxies of putative CND significantly predicted phoneme prediction accuracy from the PRP classifier and explained more than a quarter of the variance in the accuracy, jointly with neural tracking acoustic envelope and onsets. Notably, the age-group difference (middle-aged < younger) in phoneme prediction accuracy was still significant after controlling for peripheral and central processing measures.

### Neural Dedifferentiation to Speech Sounds in Middle Age

Following Khalighinejad et al. (2017) to derive PRPs, which represent cortical responses to individual phonemes, we obtained multiple lines of evidence in support of the hypothesized age-related neural dedifferentiation. The classifiers trained to predict phonemes from PRPs revealed less accurate and more uncertain predictions in middle-aged adults compared with younger adults (Figure 3A–B). Furthermore, information relevant to phoneme predictions in the PRPs was delayed (Figure 3D–H) and more distributed across electrodes (Figure 3C–E), consistent with the less specialized and more correlated activity across the brain in older adults (Du et al., 2016). Given that phoneme representations emerge from STG neural ensembles tuned to specific phonetic features, as revealed by ECoG data (Mesgarani et al., 2014), we also explored the featural relationship between phonemes in our noninvasive PRP measures. We considered the [syllabic], [sonorant], and [continuant] features, which capture manner class distinctions important to speech (e.g., vowels [+syllabic] versus consonants [−syllabic]) and modeled the relationship between phonemes at an abstract level. The representational similarity analysis revealed that such a relationship was less distinctly represented in middle-aged adults’ PRPs when including all the features (Figure 4). Note that as the stimuli were presented in quiet, the results cannot be attributed to differential effects of noise masking on the two age groups. Together, these findings suggest that cortical representations of phonemes and the features that comprise them are less differentiated, or “fuzzier,” in middle-aged listeners. The current study not only replicated the neural dedifferentiation (Park et al., 2004) in the context of meaningful, naturally produced speech sound units, but also offered novel evidence that such deficits may emerge as early as in middle age.

The fuzzier, delayed, and more distributed network of phoneme representations may have significant downstream speech perceptual consequences. Age-related neural dedifferentiation has been linked to slower speed with which individuals make judgments about the perceived stimuli (Park et al., 2002; Salthouse, 1996). This coincides with the relatively delayed relevance peak of our middle-aged participants, suggesting that they may show slower behavioral discrimination of phonemes. Even when no explicit judgments about phonemes were required and coarse comprehension performance did not differ, the fuzzier and more dispersed phoneme representation network was likely to induce subtle challenges to word recognition and more effortful listening. Indeed, middle-aged adults demonstrated poorer words-in-noise perception, consistent with past work reporting similar speech perceptual declines for middle-aged adults, regardless of audibility (Helfer & Jesse, 2021). In response to the reduced phonemic distinctiveness, listeners may allocate more cognitive resources in discriminating phonemes during spoken language processing. Per the ease of language understanding model (Rönnberg et al., 2013), the additional resources required for listening may allow for less cognitive allocation for performing a concurrent task or achieving a higher-level reasoning of the discourse. In fact, some accounts, such as the information degradation hypotheses (Monge & Madden, 2016), have attributed the chronic changes to resource allocation as having the potential to increase the risk of cognitive decline.

Middle-aged adults sometimes subjectively report speech perceptual difficulties while exhibiting clinically normal pure-tone audiograms, leading to the perception that they are “overestimating” their difficulties (Bainbridge & Wallhagen, 2014; Demeester et al., 2012; Helfer & Jesse, 2021; Kamil et al., 2015). However, our findings challenge this view, suggesting that even without measurable hearing loss, middle-aged adults still struggle with maintaining distinct neural representations of linguistically relevant units bearing critical information during spoken language processing. Recent studies have also shown that PRPs can reveal differences in neural encoding of phonemes between normal-hearing individuals and those with cochlear implants (Aldag & Nogueira, 2024), highlighting their potential as a promising noninvasive marker for detecting subtle speech perception challenges. Although practical considerations and further development are necessary, incorporating PRPs into clinical protocols, alongside standard audiograms and other peripheral measures, could capture central decline in the representations of linguistically meaningful speech sounds.

Beyond clinical diagnostics, a key question raised by our results is the underlying mechanism(s) driving cortical dedifferentiation in middle-aged adults. Currently, we speculate that the reduction in cortical specialization to phonemes reflects an age-related decline in inhibitory neurotransmitters in the cortex (Zuppichini et al., 2024). According to the computational model proposed by Li and colleagues (Li et al., 2001; Li & Rieckmann, 2014), cognitive aging and neural dedifferentiation are linked to age-related changes to the availability of neurotransmitters. Specifically, decreases in neurotransmitter availability can reduce inhibitory control and increase noise in the information processing of cortical neurons, which reduces the distinctiveness and fidelity of neural representations. Compared with younger adults, middle-aged and older adults are expected to demonstrate neural dedifferentiation given myriad evidence from positron emission tomography revealing pervasive decline in markers of neurotransmitter activity as a function of age across adulthood (Kaasinen & Rinne, 2002; Suhara et al., 1991; Volkow et al., 1996). Studies in macaque models have noted dramatic age-related changes in the response patterns of the auditory cortex, such as increased spatial tuning and reduced temporal fidelity of response, which suggest a destabilized balance of neuronal excitation and inhibition (Recanzone, 2018). One specific driver of this imbalance could be lower levels of GABA, which reduces inhibitory control in the central auditory system and causes central gain, or maladaptive amplification of cortical responses (Burianova et al., 2009; Stebbings et al., 2016). The hyperexcitability of neurons due to such gain has been observed in older adults compared with younger adults (Hermans et al., 2018) and can lead to noisier, less distinct cortical representations that disrupt downstream speech perception (Dobri & Ross, 2021; Lalwani et al., 2019).

Indeed, it is likely that the neural dedifferentiation of linguistically relevant speech units—and the presumed cortical noise contributing to it—are inherited at least partly from subclinical peripheral deficits, particularly as a sequela of CND. Animal studies in mice offer direct evidence that CND increases internal noise in the auditory cortex, resulting in poorer behavioral detection of tones (Resnik & Polley, 2021) and discrimination of speech sounds (Monaghan et al., 2020). In fact, central gain due to reduced GABA levels in older adults can arise out of the need to compensate for degraded afferent input due to auditory nerve atrophy (Harris et al., 2022). Thus, there may exist a relationship between CND and cortical dedifferentiation of phonemes, which is partially supported by our data.

### Neural Dedifferentiation of Phonemes Is Associated With Putative CND, But Peripheral and Acoustic Predictors Only Explain a Third of the Variance

We observed that measures of putative CND were indeed associated with phoneme dedifferentiation. While CND cannot be verified in humans in vivo, the ABR wave I amplitude reflects the integrity of the peripheral auditory nerve, with reduced amplitudes indicating putative CND (Furman et al., 2013; Hall, 2006; Hood, 1998; Kujawa & Liberman, 2009, 2015; Sergeyenko et al., 2013). It is also argued that elevated thresholds at EHF (>8 kHz) could indicate higher risk for CND (Liberman et al., 2016; Lough & Plack, 2022) and predict performance on speech perception in noise (Badri et al., 2011; Yeend et al., 2019), although some (Helfer et al., 2024) found no evidence for such predictive power after controlling for PTA at conventional speech frequencies. Our middle-aged adults did show reduced wave I amplitudes and higher EHF thresholds (Figure 6A–D), despite normal audiograms, suggesting some evidence for putative CND. However, subclinical differences in hearing sensitivity across conventional speech frequencies (pure tone averages across 1, 2, and 4 kHz) were observed between young and middle-aged adults. When controlling for PTA, wave I amplitude differences between age groups did not remain, suggesting the decline in wave I amplitudes observed in middle-aged adults were not exclusively caused by CND, but other mechanisms of peripheral decline as well. Such findings highlight the challenge of isolating the impact distinct peripheral mechanisms have on higher auditory processes. Here, we addressed this challenge by extending our analysis to examine the relationship between cortical representations of speech and the multitude of factors at play in the aging auditory system.

Our stepwise regression analyses showed that lower ABR wave I amplitudes were significantly related to lower phoneme prediction accuracy from the PRP classifier. Notably, our regression model initially included PTA and EHF thresholds, and neither revealed multicollinearity with wave I amplitudes. Thus, while subclinical differences in hearing sensitivity impacted age-group differences in wave I amplitudes, hearing sensitivity did not relate to wave I amplitudes when assessed on a continuous spectrum. Furthermore, the regression analysis showed that neither PTA nor EHF thresholds were significant factors for phoneme decoding accuracy, while wave I amplitudes were. As such, while we acknowledge that multiple peripheral factors are at play in the aging auditory system, these results leave room for the possibility that higher auditory processes could be impacted by putative CND, as proxied by wave I amplitudes, and not by changes in hearing sensitivity alone. With these findings and prior studies showing that central gain arises to compensate for peripheral deficits (Harris et al., 2022), we posit that similar compensatory mechanisms may be at play in middle-aged adults. That is, to adapt to degraded peripheral inputs induced by putative CND, disinhibition may occur throughout the central auditory system with the goal of increasing the neural gain. This is evidenced in our results through the normalization of wave V amplitudes despite lower wave I amplitudes in middle-aged adults. Indeed, our secondary regression analyses revealed that ABR wave V/I amplitude ratio accounted for some variance in cortical representations of phonemes, suggesting central gain compensation is, to some extent, a factor in the central mechanisms involved in speech perception deficits. While such adaptation reflects central plasticity, it may come at the cost of elevated cortical noise leading to neural dedifferentiation and poorer speech sound discriminability (Monaghan et al., 2020). Here, we further demonstrated that such costs include less differentiated phoneme representations.

In addition to markers of putative CND, the stepwise regression revealed that other central process factors, specifically better neural tracking of acoustic onsets, were also associated with higher phoneme prediction accuracy. This association was compatible with the privileged role of onset information in cortical processing and speech recognition. Not only is the auditory cortex sensitive to acoustic onsets in speech (Daube et al., 2019; Hamilton et al., 2018), but the precise spatiotemporal patterns of cortical responses within milliseconds of a consonant onset robustly predict behavioral discrimination of the consonant (Engineer et al., 2008). Computational models and behavioral data from the spoken word recognition literature also demonstrate an advantage for word-initial segments in guiding lexical access and managing lexical competition (Huettig et al., 2011; McClelland & Elman, 1986; Norris & McQueen, 2008). Although acoustic onsets do not always coincide with word onsets, these findings highlight the importance of onset information in speech processing. Consequently, individuals whose brains are more capable of tracking acoustic onsets tend to retain more distinct phoneme representations.

Nevertheless, even after correcting for the effects of peripheral (wave I amplitudes) and central (ABR wave V/I ratio, neural tracking of acoustic envelope and onsets) factors, the residual analyses showed that middle-aged adults’ lower phoneme prediction accuracy was still significantly different from young adults. The remaining accuracy gap between the two age groups raises the possibility that other central factors of neural dedifferentiation exist that occur *independently* of peripheral changes and may relate proximally to speech perception. Future work integrating PRP, peripheral deficit measures, and concentrations of neurotransmitters in the auditory cortex could aid in uncovering these factors, particularly in an animal model.

### Limitations

The mechanisms regarding neural dedifferentiation as discussed above are speculative, as we cannot verify that the PRPs derived from EEG relate directly to changes in the auditory cortex and especially the STG. However, the findings offer a few intriguing avenues for further research. These include examining age-related changes in phoneme or feature selectivity of neural responses in the STG using recording techniques with high spatial resolution. As mentioned, ECoG studies indicate that local sites in the STG tune to specific phonetic features (Hamilton et al., 2021; Yi et al., 2019). The degree of selective tuning can be quantified with measures such as the phoneme selectivity index (Mesgarani et al., 2014) and the neural dedifferentiation hypothesis would then predict that such indices should decrease as a function of age. Furthermore, given the phonological theories of distinctive features (Chomsky & Halle, 1968) and the results from the representational similarity analysis (Figure 4), it may also be expected that age-related neural dedifferentiation would first affect finer featural distinctions lower in the phonological feature hierarchy, such as place distinctions, as compared with manner distinctions. Addressing these predictions could contribute to a more nuanced understanding of how aging relates to cortical changes in the central auditory system.

### Conclusion

In summary, we demonstrate that the neural distinctiveness of speech representations is reduced in middle-aged adults, who also demonstrate signatures of putative CND and speech perceptual deficits despite normal audiograms. While processing naturalistic speech, middle-aged adults show reduced speech sound distinction and featural relationships, delayed timing, more uncertainty, and more distributed processing relative to younger adults. These age-related changes cannot be entirely attributed to peripheral factors, such as putative CND or lower-level acoustic processing. Fuzzier processing of the basic unit of spoken language processing may have significant downstream consequences on speech perception, such as elevated listening effort and greater need for cognitive-linguistic resources to scaffold spoken language processing. These findings highlight the need to consider age-related changes in cortical representations of critical speech sound categories and features when exploring the sources of speech perception challenges in middle-age.

## ACKNOWLEDGMENTS

This research used the resources provided by the Quest high-performance computing cluster at Northwestern University and the University of Pittsburgh Center for Research Computing, RRID:SCR_022735 (NSF OAC-2117681). The authors would like to thank Sarah Anthony, Olivia Flemm, Claire Mitchell, and Leslie Zhen for their assistance with participant recruitment and data collection.

## FUNDING INFORMATION

Jacie McHaney, National Institutes of Health (https://dx.doi.org/10.13039/100000002), Award ID: F31DC020085. Aravindakshan Parthasarathy, National Institutes of Health (https://dx.doi.org/10.13039/100000002), Award ID: R21DC018882. Bharath Chandrasekaran, National Institutes of Health (https://dx.doi.org/10.13039/100000002), Award ID: R01DC013315.

## AUTHOR CONTRIBUTIONS

**Zhe-chen Guo**: Conceptualization; Data curation; Formal analysis; Software; Validation; Visualization; Writing – original draft. **Jacie R. McHaney**: Conceptualization; Data curation; Formal analysis; Funding acquisition; Investigation; Methodology; Project administration; Software; Validation; Visualization; Writing – original draft. **Aravindakshan Parthasarathy**: Conceptualization; Funding acquisition; Project administration; Resources; Supervision; Writing – review & editing. **Kailyn A. McFarlane**: Project administration; Writing – review & editing. **Bharath Chandrasekaran**: Conceptualization; Funding acquisition; Project administration; Resources; Supervision; Writing – review & editing.

## DATA AND CODE AVAILABILITY STATEMENT

The data produced during this study and the code used to analyze are available on the Open Science Framework at https://osf.io/jqf8t.

## Supplementary Material



## TECHNICAL TERMS

Cochlear neural degeneration (CND): Loss of afferent synapses between inner hair cells in the cochlea and auditory nerve fibers, often due to aging or noise exposure.
Neural dedifferentiation: A reduction in the distinctiveness of neural representations, characterized by less specialized, more correlated cortical activity patterns.
Phoneme-related potential (PRP): Event-related potentials time-locked to phoneme instances in speech, averaged across multiple occurrences to capture phoneme-specific cortical responses.
