# Supplementary material for: Reduced Neural Distinctiveness of Speech Representations in the Middle-Aged Brain
Source: Neurobiol Lang (Camb). 2025 Jun 18;6:nol_a_00169. doi: 10.1162/nol_a_00169 (PMC12327429; doi:10.1162/nol_a_00169)
Supplement: Supplementary file 1 [file nol-6-1-169-s001.pdf]

Supplementary Materials

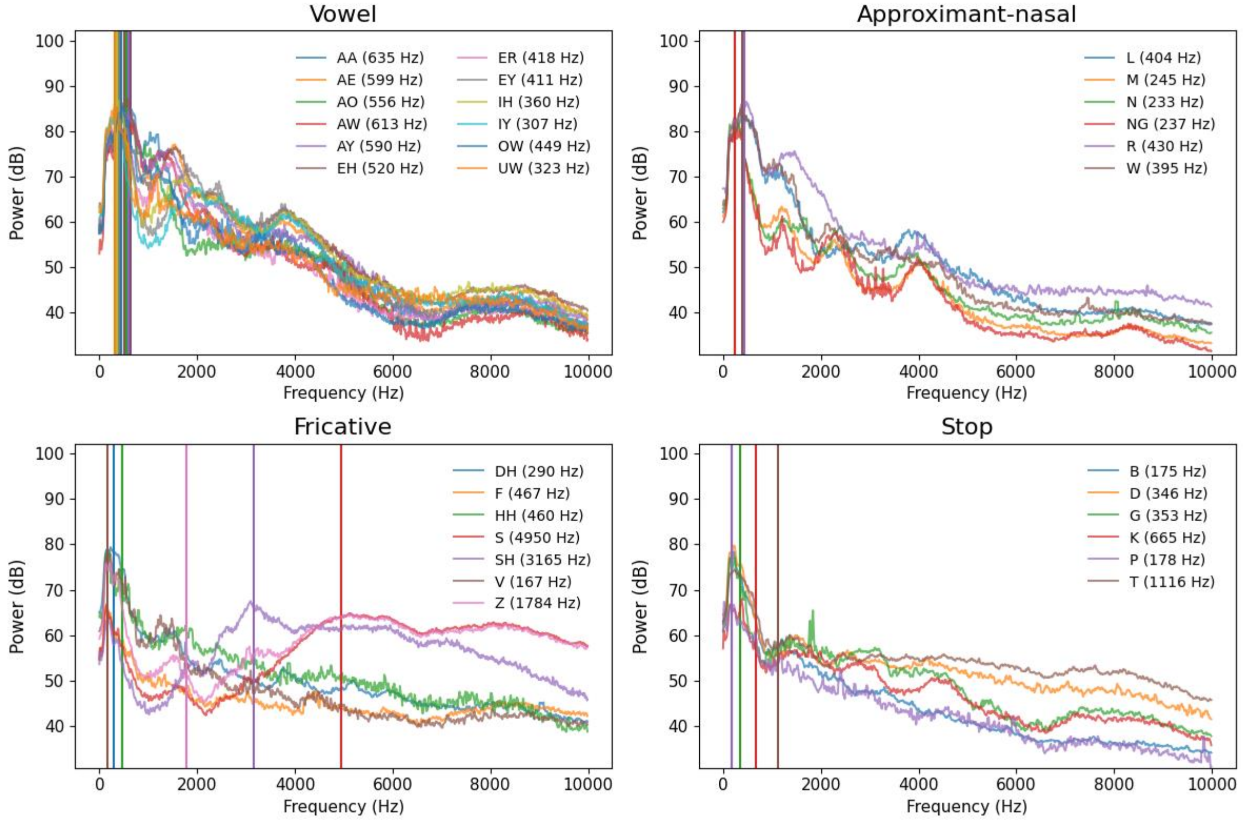

Figure S1. Average power spectrum up to 10 kHz for each phoneme in the *Alice's Adventures in Wonderland* audiobook. The vertical lines mark the average spectral peak frequency for each phoneme, with the frequency value shown in parentheses within the legend. Phonemes are grouped based on manner of articulation.
